# High-resolution mapping and dispersion analyses of volcanic ballistics emitted during the 3rd July 2019 paroxysm at Stromboli

**DOI:** 10.1038/s41598-023-39600-w

**Published:** 2023-08-18

**Authors:** M. Bisson, C. Spinetti, R. Gianardi, K. Strehlow, E. De Beni, P. Landi

**Affiliations:** 1 Istituto Nazionale di Geofisica e Vulcanologia - Sezione di Pisa, Pisa, Italy; 2 Istituto Nazionale di Geofisica e Vulcanologia - ONT, Rome, Italy; 3 GEOMAR Helmholtz Centre for Ocean Research Kiel, Kiel, Germany; 4 Istituto Nazionale di Geofisica e Vulcanologia - Osservatorio Etneo, Sezione di Catania, Catania, Italy; 5 Present Address: Mitiga Solutions SL, Barcelona, Spain

**Keywords:** Natural hazards, Solid Earth sciences

## Abstract

A detailed mapping of volcanic ballistic projectiles emplaced in a defined area, represents the starting point to derive preparatory data in hazard and risk studies of ballistics phenomena. Considering as case study the 3rd July 2019 paroxysmal eruption occurred at Stromboli volcano, we map and analyse at very high spatial resolution (8 cm) the distribution of the ballistic spatter clasts emplaced on the E flank of the volcano. The resulting map identifies and reproduces as geospatial polygon elements 152,228 spatter clasts with areal dimensions from 0.03 to 4.23 m^2^. Dispersed on 0.407 km^2^, the spatters cover an area of 29,000 m^2^ corresponding to an erupted products volume from 2.3 to 7.0 × 10^3^ m^3^, calculated here for the first time. Spatial analyses indicate that the area mostly affected by the clasts emplacement is between N67.5 and N135 directions, identifying a preferential deposition between N112.50 and N123.75 directions. The clasts size distribution rapidly decreases with the size increase, highlighting a nearly constant ratio small/large clasts regardless the distance from the vent. Finally, additional investigations reveal that clasts dispersion parameters decrease progressively with the distance from the vent only along one direction (N67.5), highlighting how the morphology influences the deposition and remobilisation of mapped ballistics.

## Introduction

Ballistic projectiles are centimetre to meter-sized clasts, both solid and molten, which are commonly ejected during explosive eruptions and move in the atmosphere along ballistic trajectories. They have exit velocities ranging from tens to hundreds of m/s and can travel up to ten kilometres from the vent, although they are usually restricted within 5 km^1–5^. Ballistics are associated with all forms of explosive eruptions but are considered major hazards of phreatic, phreatomagmatic, vulcanian and strombolian eruptions and are the most common cause of fatal incidents in the vicinity of volcanic vents at many volcanoes in the world^2,6,7^. A useful tool for supporting the risk management of ballistics is the production of hazard maps^7^ that require, as indispensable data, the geo-spatial mapping of the ballistics emplaced during explosive events of different magnitudes. Nevertheless, few studies are addressed reproducing the geo-spatial dispersion of ballistics and, especially, do not re-build the 2D geo-spatial geometry of the ballistics emplaced on the ground. Most of the published works have mapped the ballistics distribution reconstructing isopleths derived from their counting and size, obtained from direct observation in the field, or individuated from images acquired by a photographic camera mounted on aerial platforms^8–14^. At Stromboli volcano, the most recent studies reconstruct the boundary of the area affected by ballistic fallout that occurred during the major explosions and paroxysms by field surveys carried out within a few days after the eruption, when the newly erupted material was still well recognizable^9,15–18^. However, it is a very time-consuming approach forcing operators to stay in dangerous areas for a long time and, if some sectors of the volcano are inaccessible, this may prevent a complete reconstruction of the areal distribution. The field approach might lead to failure to collect enough measurements that are preparatory data to develop subsequent robust probabilistic hazard maps. In this work, we present for the first time a very high spatial resolution mapping of the ballistics emplaced on the eastern summit flank of Stromboli volcano during the 3rd July 2019 paroxysm. In detail, we consider the spatter clasts emplaced during the early phases of the eruption. The spatters represent the molten ballistics ejected from the vent, re-shaped in-flight, and flattened on impact with the ground, involving strong shortening of vertical and lengthening of horizontal axes.

By using a GIS-based methodological approach combined with high-resolution drone images elaborations, we map the spatter clasts through geo-coded polygonal elements, which reconstruct the boundary of each spatter clast. This methodological approach allowed us, rapidly, to analyse the ballistic spatter clasts dispersion exploiting a large amount of data that is impossible to obtain in a reasonable time with classic field surveys. We derive the total extent of spatter clasts associated with an estimate of the relative volume, the dispersion parameters with their variation moving away from the vent, and the clasts size distribution. Finally, we discuss the obtained results highlighting how the terrain morphology influences the clasts deposition and remobilization.

### Stromboli and the 3rd July 2019 paroxysm

**Figure 1:**
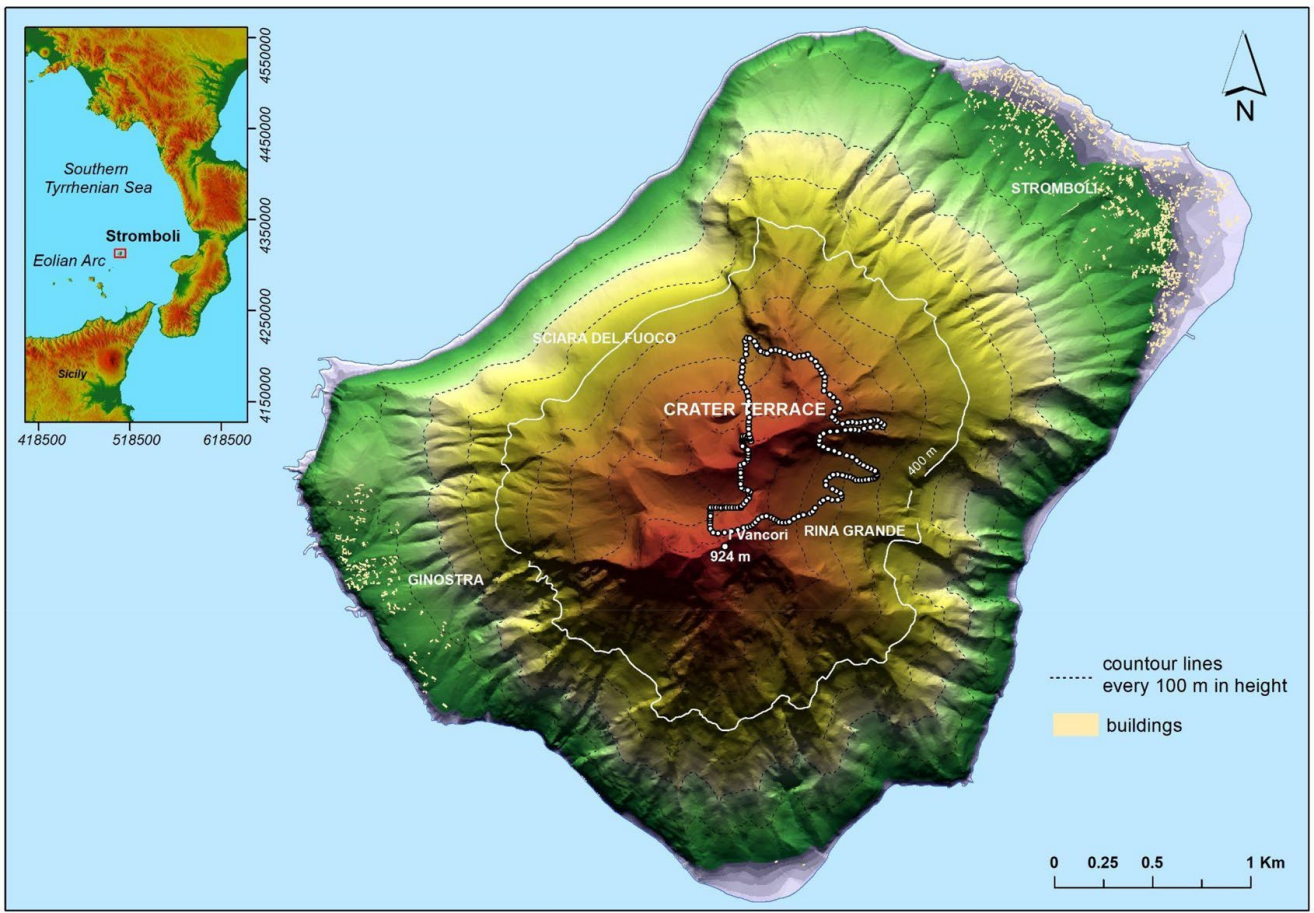
2012 Lidar DEM of Stromboli superimposed on the shaded relief image. The dotted white line delineates the boundary of the study area. The white contour represents the maximum height accessible to hikers after the events of 2019. The inset shows the geographical setting of Stromboli Island, the coordinates are reported in metres according to the WGS84 UTM 33N geodetic cartographic reference system. (Data sources of the DEM were provided under the INGV-DPC agreement). The map was composed in ArcGIS 10.5 software (ESRI Platform).

Stromboli, the northernmost volcanic island of the Aeolian Arc (Southern Italy), has an elongated shape of ~ 3 × 4 km with maximum altitude of 924 m a.s.l. (Fig. 1). It presents an inaccessible horseshoe-shaped depression on the N flank (Sciara del Fuoco), resulting from several flank collapses^19,20^. The activity occurs in the crater terrace (Fig. 1) located in the upper part of the Sciara del Fuoco (~ 750 m a.s.l), where several active vents are always present^17,21–23^. The normal activity of the volcano consists of mild strombolian explosions with a frequency from 5 to 30 explosions per hour, ejecting lapilli and bombs to heights of a few hundreds meters above the vent and up to a few tens of meters beyond the edges of the crater terrace, usually rolling down along the Sciara del Fuoco^17,24^. The mild activity is occasionally interrupted by energetic, impulsive explosions, lasting tens of seconds to a few minutes, named major explosions and paroxysms, depending on the magnitude and intensity of the event^17,25^. These energetic explosions form eruptive columns that in the paroxysms can reach heights of several km. Considering the past 140 years, the frequency of major explosions and paroxysms is typically in the range of 0–6 and 0–4 per year, respectively, paroxysms being much less common than major explosions^26^. While, in the last 23 years 4 paroxysms have occurred and the frequency of major explosions has been 2–3 per year.

During the major explosions, ballistic juvenile and lithic bombs fall in the upper portion of the volcano, and represent the main hazardous phenomena, occasionally associated with wildfire started by the fallout of incandescent material on dry vegetation. While, volcanic hazards associated with the paroxysms comprise a variety of phenomena including: ballistic fallout that often affect Stromboli and Ginostra villages, located on the NE and SW coast of the island, respectively at 2.1 and 1.8 km from the vent; tephra fallout; pyroclastic density current (PDC) induced by gravitational instability of hot material accumulated on the upper part of the volcano during the eruption; pyroclastic flows within the Sciara del Fuoco (Fig. 1); tsunamis triggered when the pyroclastic flows enter into the sea; shockwaves; widespread wildfires^17,18,27^. In detail, PDCs represent highly dangerous phenomena associated with the paroxysms, but are most likely confined within the canyons and valleys. On the contrary, the ballistics might reach every point of the island and thus they can represent one of the main hazards.

**Figure 2:**
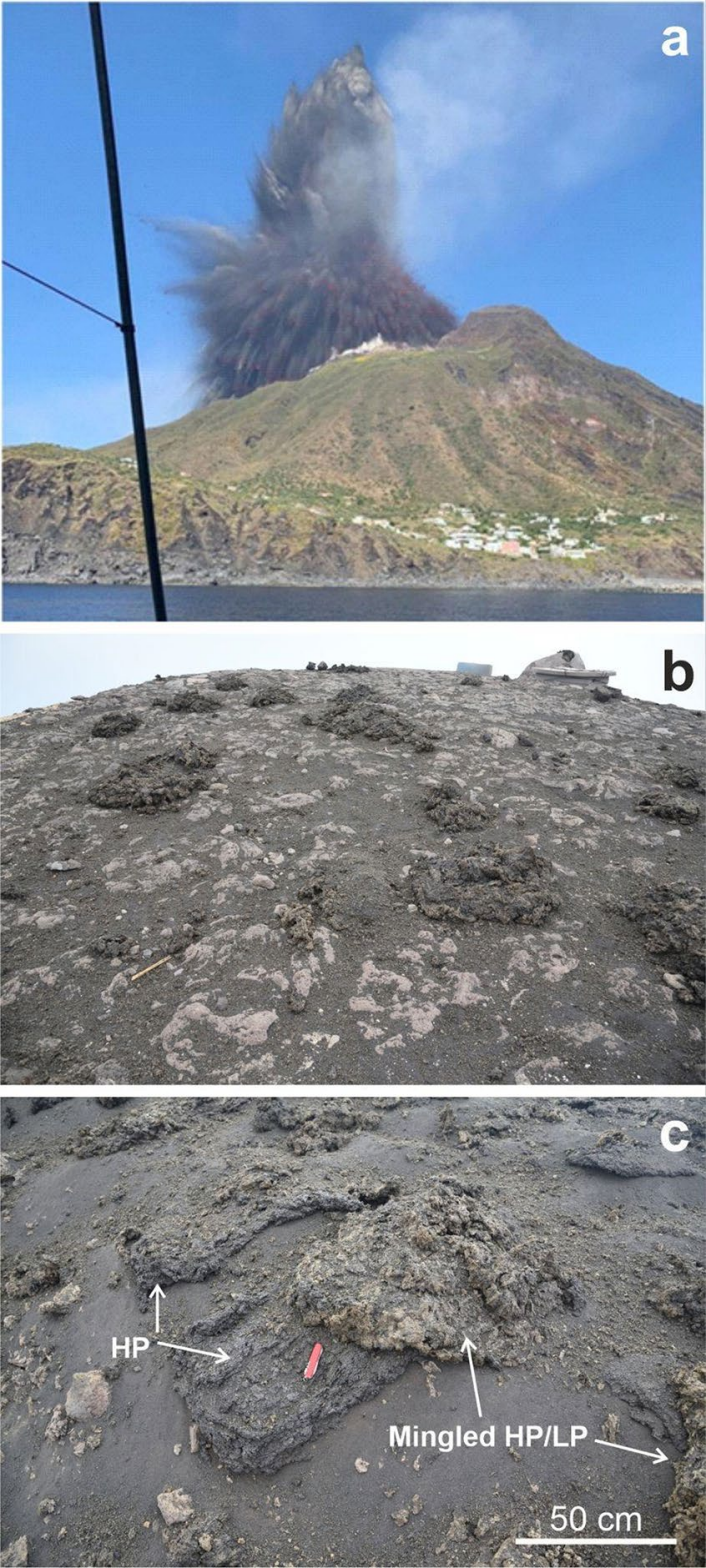
The early phase of the 3rd July 2019 paroxysm and the spatter clasts ejected. (**a**) The photo was taken from a sailing boat near Ginostra, a few seconds after the onset of the explosion (modified from Giordano and De Astis, 2021); (**b**) spatter clasts on the helipad located at 850 m a.s.l.; (**c**) in the centre of the image two partially overlapping spatter clasts are shown. The two different colours correspond to HP and mingled HP-LP spatters.

The last 2 paroxysms, which occurred during the volcanic crisis of 2019, are dated on 3rd July and the 28th of August. The 3rd July paroxysm started with two sudden explosions, with cryptic precursors, forming a nearly spherical bulb above the crater terrace^18^ (Fig. 2a). The bulb, initially expanded radially and then slightly asymmetric seaward, ejected ballistic molten projectiles producing the fallout of decametric to metric juvenile clasts, associated with minor quantities of lithic lava blocks which constitute about 1–5 vol% of the deposit in the summit area^17^. Juvenile clasts (Fig. 2b,c) consist of both scoriaceous, dark-grey, crystal-rich spatters (HP = Highly Porphyritic) and pumiceous/scoriaceous, yellowish spatters resulting from mingling processes between shallow, volatile-poor HP magmas and volatile-rich Low Porphyritic (LP) magmas rising from depth^28,29^. During this early phase of the eruption, ballistics were launched down the volcano’s slope as low as 500 m a.s.l. along the N flank above Stromboli village, and below 200 m a.s.l. towards Ginostra village, triggering fires, and reaching the sea in front of the Sciara del Fuoco. According to Giordano and De Astis^18^, the ballistics have formed a continuous spatter coverage within 500 m from the vent in the SW direction and 350 m in the NE direction. At greater distances from the vent, the spatter coverage becomes discontinuous and the areas are affected by scattered spatter clasts. This phase was followed by the development of an eruptive column that rose to 6–8 km high^18,30,31^ dispersing ash, lapilli and bombs in the SW sector of the island. Two pyroclastic flows descended into the Sciara del Fuoco, triggering small tsunamis at sea entrance, while at least two gravity-induced flows affected the summit. In particular, a flow descended the SE flank of the volcano through Rina Grande^30^ and a glowing avalanche in the N flank^18^. Fires lasting days involved large areas of the island generating heavy environmental impacts concerning endemic vegetation loss, damages to agricultural heritage, and transformations to landscape patterns^32^.

In this work, we investigate the volcano E sector (Fig. 1). In accordance with what described in literature^18^, such sector was only affected by spatter clasts and a minor quantity of lithic blocks, both emitted during the early phase of the 3rd July 2019 eruption. Tephra fallout (ash, pumiceous and scoriaceous lapilli and bombs) associated with the column phase^18^, instead, affected the SW sector, well as spatter clasts and lithic bombs.

## Results

### High-resolution mapping of spatter clasts

**Figure 3:**
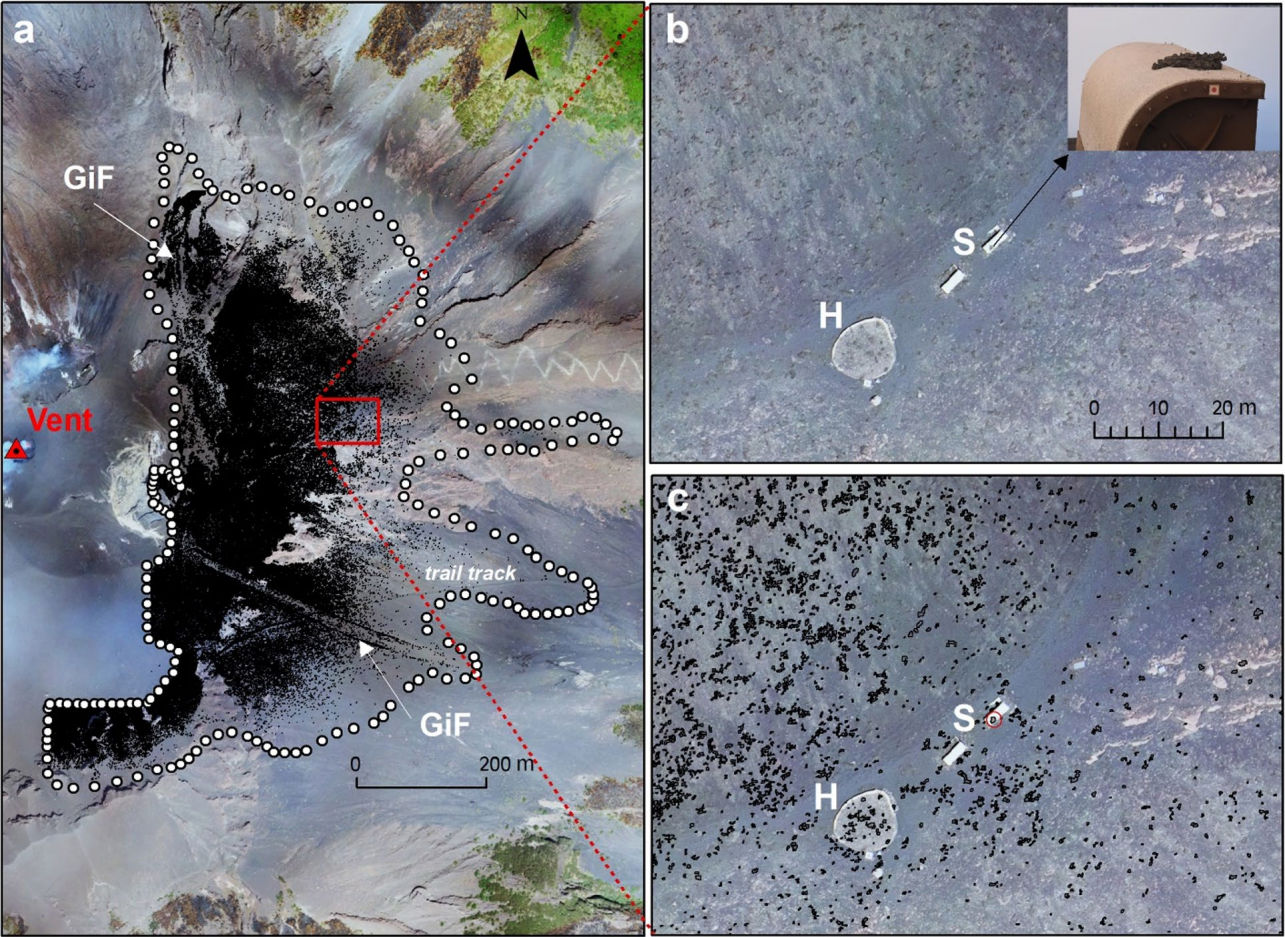
(**a**) The spatter clasts digital mapping overlain to Drone imagery (see Methodology Section). The Vent is positioned in the SW sector of the crater terrace, according to what is reported in references^17,23^ (GiF: Gravity induced Flow); (**b**–**c**) zoom of the East area of the Vent (red inset in **a**), before (**b**) and after (**c**) the elaboration. The helipad (H) and the shelters (S) are located at 850 m a.s.l. The inset in (**b**) displays the photograph of the spatter clast emplaced on the shelter roof. In (**c**) the spatter clasts are reproduced as polygon elements (in black). The red circle surrounds the spatter clast on the shelter roof. The maps were composed in ArcGIS 10.5 software (ESRI Platform).

The study area covers 0.407 km^2^ and is localised in the E flank of Stromboli at an altitude above 500 m a.s.l. (Fig. 1). It is delineated by a white dotted line (Fig. 3a) that corresponds to a real limit of the spatter clast fallout only towards N, S and E. Whereas, at W, the dotted line corresponds to the limit beyond which it is not possible to clearly distinguish the spatter clasts due to the presence of the volcanic plume that, in this zone, reduces the visibility of the ground^33^. In the study area, 152,228 spatter clasts are mapped with dimensions ranging from the minimum detectable area of 0.03 m^2^ to a maximum of 4.23 m^2^. They cover a total area of 29,000 m^2^ extended between NE and SE directions and from a minimum distance from the vent of 250 m to a maximum distance of 950 m, defining two lobes directed towards E and SE, respectively (Fig. 3a). The high spatial resolution of the map allowed to identify two Gravity induced Flows (GiF). In the S sector of the map, the GiF which descended the SE flank is identified thanks to a dense accumulation of spatter clasts on lateral edges (levees). This accumulation create very straight edges for ~ 450–475 m in length, and a paucity of clasts between the edges themselves. In the final portion of the GiF, the two lateral edges converge into a very narrow frontal lobe, where the spatter clasts accumulation (clasts with area > 0.03 m^2^) is not detected, as also reported in^30^. The GiF starts from 890 m a.s.l., goes straight reaching a maximum width of 21 m at 710 m a.s.l, and stops at 599 m a.s.l. with a very narrow frontal lobe. The total length of the GiF is 500 m. These observations, in particular the dense lateral accumulation of the spatter clasts and their paucity between the edges, could suggest a possible post-emplacement sliding phenomenon. Similarly, also in the N sector of the map (Fig. 3a), a second GiF is identified. Such flow, with a length of 465 m, starts from 898 m a.s.l and stops at 615 m a.s.l. and its width ranges from 28 to 80 m. With respect to the previous one, this GiF is characterized by (1) more curved edges where the spatters are accumulated and (2) a larger amount of clasts between the edges, especially in the final portion of the flow where several accumulation lobes are better defined. These evidences could suggest a different rheological behaviour of the flow (e.g. due to a higher emplacement temperature) or that the total volume of remobilised material controls the final morphology of the deposit. Figure 3b,c show, at a greater scale (1:1000), the zone of the helipad and shelters positioned at 850 m a.s.l. (red inset Fig. 3a) before and after the elaboration, respectively. The spatter clasts emplaced on the helipad and in surrounding areas (Fig. 3b) are reproduced as geo-spatial polygon elements (Fig. 3c). In particular, the spatter clast emplaced on the roof of the shelter (inset Fig. 3b) is well detected in our map and results to have dimensions of 85 × 70 cm (red circle Fig. 3c). Since a field survey dedicated to validating our map was not possible, the dimensions and the number of the mapped spatter clasts have been compared with those published in recent works^18,30^. Such comparison is referred to the spatter clasts on the helipad area (70 m^2^) located at 850 m a.s.l. (Fig. 3b,c). We identify 26 clasts whose major axes range from 35 to 122 cm and, among these 26 clasts, 21 clasts show a major axis greater than 40 cm. Considering the recent works^18,30^, 19 clasts with the major axis greater than 40 cm are identified in^30^, and 22 clasts whose maximum diameter is 120 cm are identified in^18^. This maximum diameter is quite close to that obtained by our elaborations (122 cm). These comparisons show a good agreement between our results and the field data considering the very irregular shape of the clasts and different times of observation. From this comparison, we associate 10% of relative error on the clasts number.

### Spatter clasts dispersion

**Figure 4:**
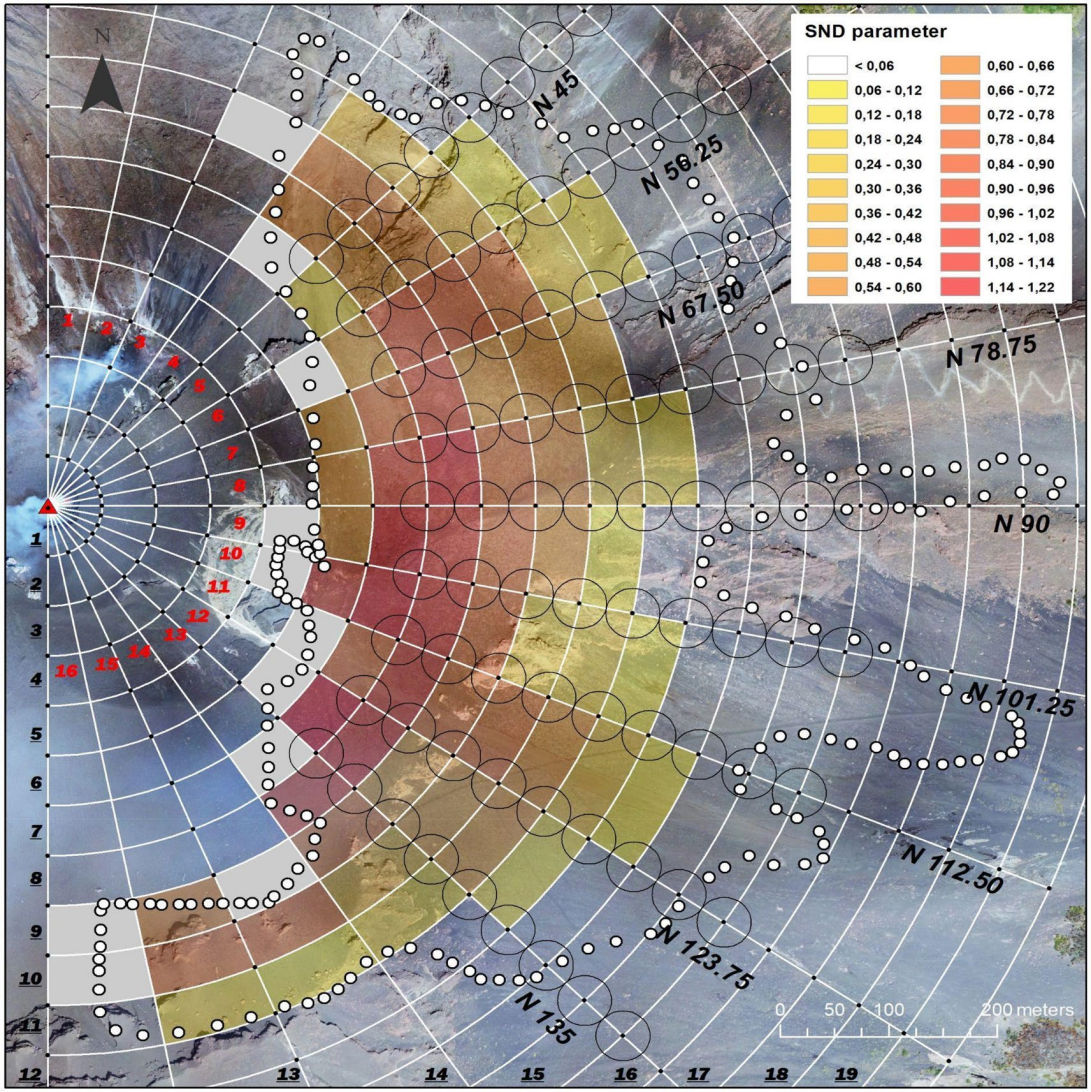
Geometry framework used for the analyses and including rings, sectors and reference elements. The number in bold black indicates the ring, and the number in bold red indicates the angular sector. The black circles (25 m of radius) represent the reference areas for analysing the variation of dispersion parameters along radial directions (white line). The different colours represent the SND parameter value calculated in each reference element (see legend). The reference elements coloured in light grey are not analysed as not entirely included in the study area (white dotted line). In the background the Drone imagery. The map was composed in ArcGIS 10.5 software (ESRI Platform).

The analyses on the spatter clasts dispersion have been obtained by investigating the two dispersion parameters, spatter number density (SND) and spatter coverage in percentage (SCP). Considering that the mapped spatters are 152,228 and cover 29,000 m^2^ on the entire study area (0.407 km^2^), SND and SCP result in 0.037 n/m^2^ and 7%, respectively. Using a circular geometry as reference area for the analyses, more detailed investigations on spatters dispersion are performed. In detail, the SND and SCP parameters are derived in a semi-circular area, constructed by rotating from N to N180 a radius of 1000 m around the vent, which was positioned in the SW sector of the crater terrace (Fig. 4), as reported in^18,30^. The dispersion of mapped clasts was investigated by using a slice of fixed angular width, not to exclude any mapped clast. The semi-circular area was divided into 20 semi-rings built every 50 m from the vent and into 16 angular sectors having a width of 11.25°. The intersection between the ring (**r**) and the angular sector (**s**) identifies the reference element (**r**, **s**) used for the dispersion analysis (Fig. 4).

The total number of spatter clasts in the study area was first analysed for angular sector by summing the spatter clasts mapped in each element (**r**, **s**), within each angular sector from N45 to N135, excluding the areas at a distance < 300 m from the vent. These angular and distance constraints allow to make comparable the resulting data. The number of spatters reaches high values in the sectors between N56.25 and N135, showing a minimum of 13,303 in angular sector **7** (N67.50–N78.75) and two maximums of 15,857 in sector **8** (N78.75–N90) and 17,434 in sector **11** (N112.50–N123.75). Instead, sector **5** (from N45 to N56.25) stands out for a lower number of spatters (6904). Figure 4 shows the SND parameter calculated for each element. The greater SND values are observed in the S–E quadrant and, mainly, in the angular sectors between N78.75 and N135 (E–SE directions). Rings **7** and **8**, located respectively between 300–350 m and 350–400 m of distance from the vent, result the most affected by the spatters deposition. SND reaches the highest values (> 1.2 n/m^2^), inside the angular sectors **9**, **10** and **12** for ring **7** and, inside the angular sector **8** for ring **8**. At distances greater than 400 m from the vent we observe a progressive decrease of the parameter towards E, reaching values < 0.1 at distances greater than 600 m from the vent (ring **13**).

**Figure 5:**
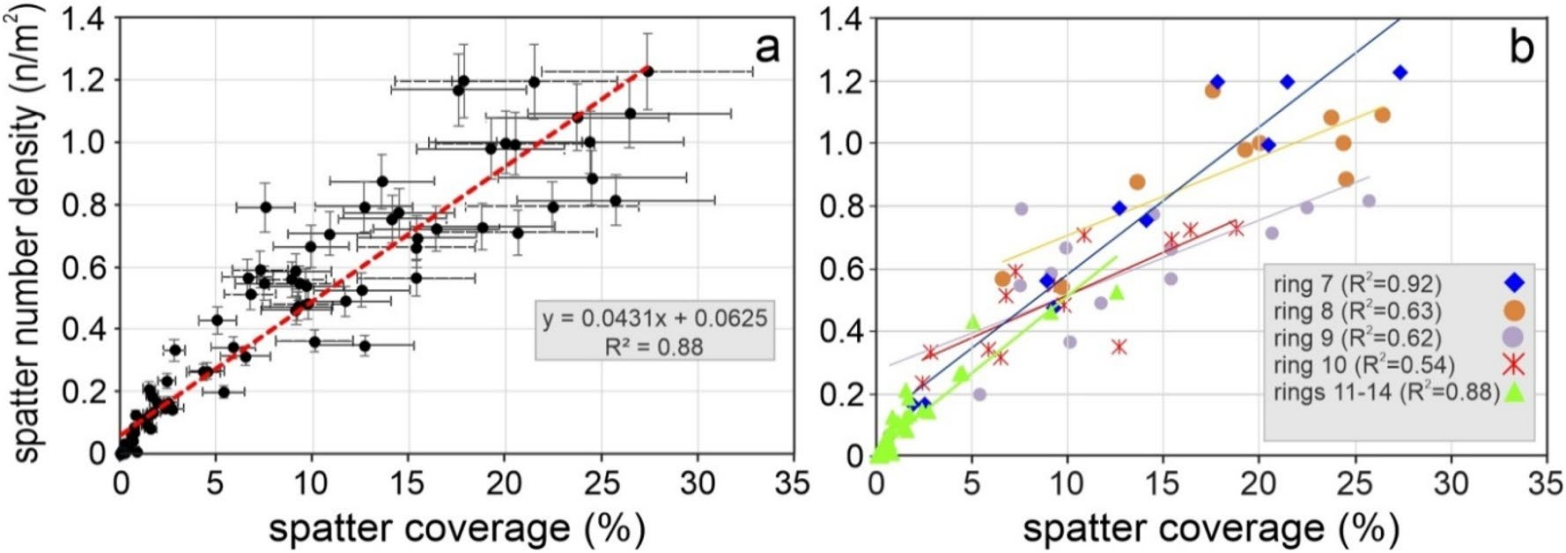
SCP vs. SND diagrams. (**a**) SCP and SND values calculated in all elements of the entire study area; (**b**) correlation between SNC and SCP inside each ring.

As a whole, SND and SCP, calculated in each element (**r**, **s**) of the entire study area, show a linear correlation with R^2^ = 0.88 (Fig. 5a). The same correlation is observed also in the ring **7** and in the rings **11**–**14** (Fig. 5b). Conversely, a poor correlation (R^2^ < 0.63) is observed in the rings **8**, **9** and **10**, where SND and SCP show a larger dispersion (Fig. 5b).

**Figure 6:**
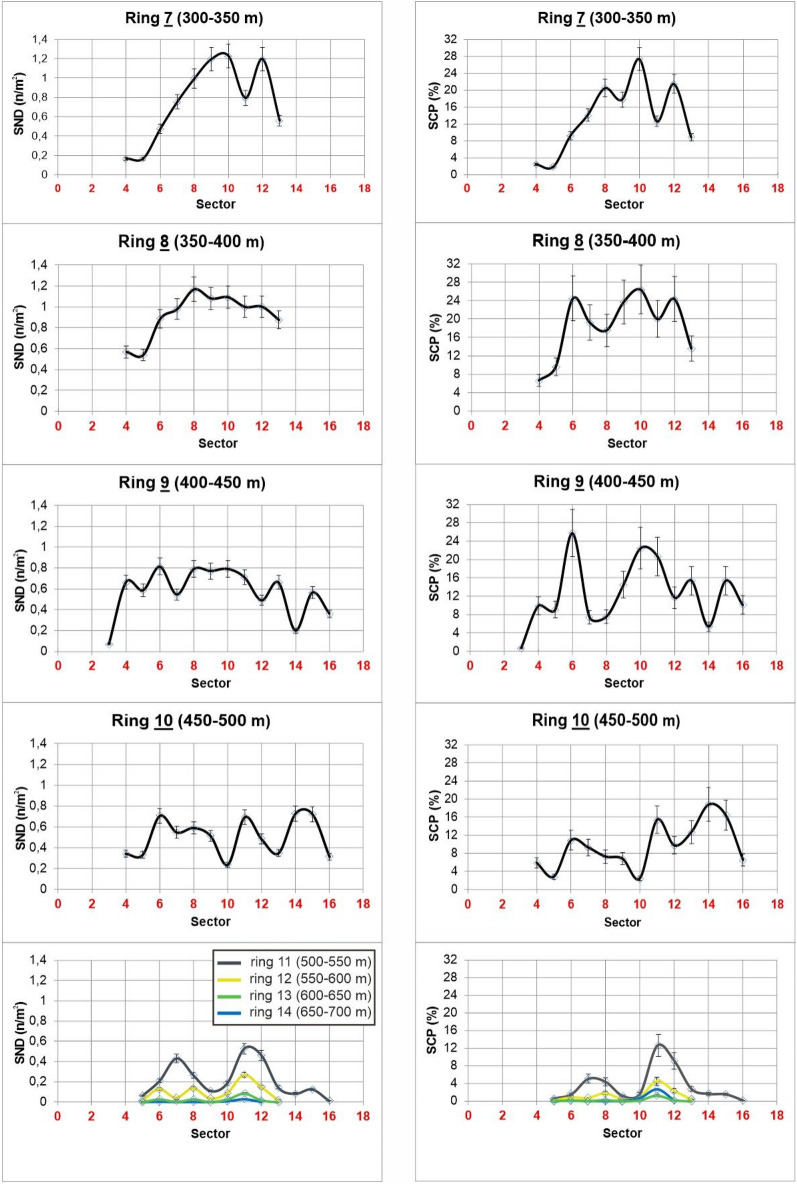
The variation SND and SCP parameters analysed for each ring, according to the angular sector.

In detail, Fig. 6 shows the trends of the two parameters within each ring, according to the angular sector. The SND variations show evident irregular trends characterised by negative or positive peaks, for example the abrupt decreasing of the SND in the element (**7**, **11**). Also, the SCP parameter shows a largely irregular distribution along each ring with evident negative and positive peaks regardless of the angular sector. In particular SCP reaches the maximum values (> 25%) in the elements (**7**, **10**), (**8**, **10**), and (**9**, **6**) and decreases below 2% at distances greater than 600 m from the vent (rings **12**–**14**). The SCP and SND variations become more regular in the outer rings (**11**–**14**; from 500 to 750 m from the vent). The different areas of the elements can slightly influence the parameters but do not invalidate the general trend.

**Figure 7:**
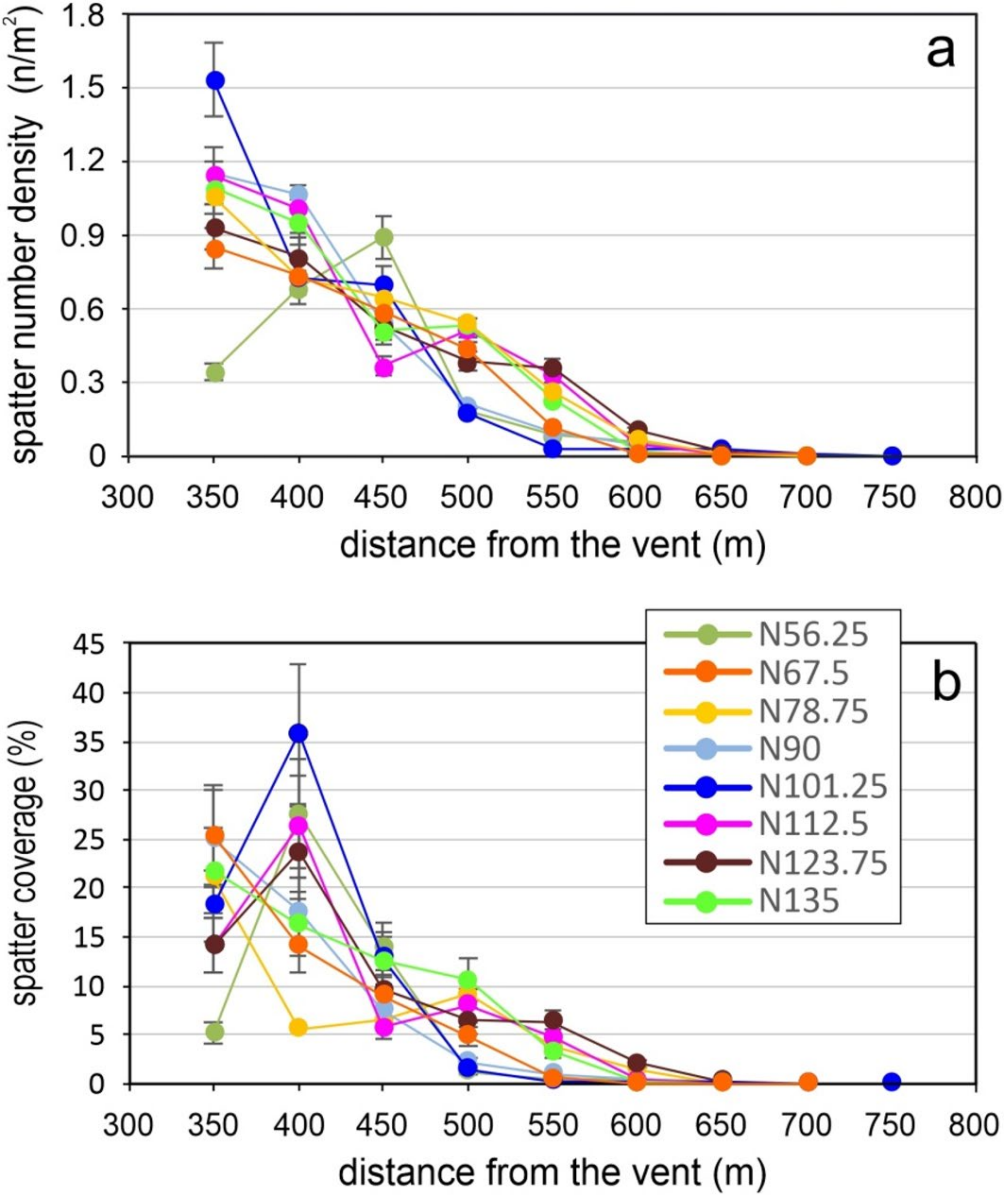
Dispersion parameters. Variation of the (**a**) spatter number density (SND) and (**b**) spatter coverage in percentage (SCP) with the distance from the vent between 350 and 750 m. The reference areas for calculating the parameters are the black circles shown in Fig. 4.

Finally, to properly investigate the variation of the parameters along the distance from the vent, we analysed SND and SCP considering the spatter clasts mapped in a fixed reference area positioned at defined distances from the vent and along different directions. For this purpose, 25 m-radius circles have been placed from the vent every 50 m, along the directions originating at the vent every 11.25°, from N56.25 to N135 (Fig. 4). Figures 7a and b show SND and SCP values plotted against the distance from the vent, respectively. To obtain consistent and comparable results, the circles positioned at a distance < 350 m and at a distance > 750 m from the vent have been excluded from the analysis for the following reasons: (i) not all circles positioned at distance < 350 m are entirely included in the study area; (ii) the distal areas (> 750 m from the vent) don’t contain detectable spatter clasts except those in the N90 direction.

The parameters SND and SCP show a general decreasing trend with the distance from the vent, but only along the N67.5 direction both parameters decrease progressively with the distance. All the other directions show spikes more or less marked (e.g. along N101.25 and N78.75 directions).

### Spatter clasts size distribution

**Figure 8:**
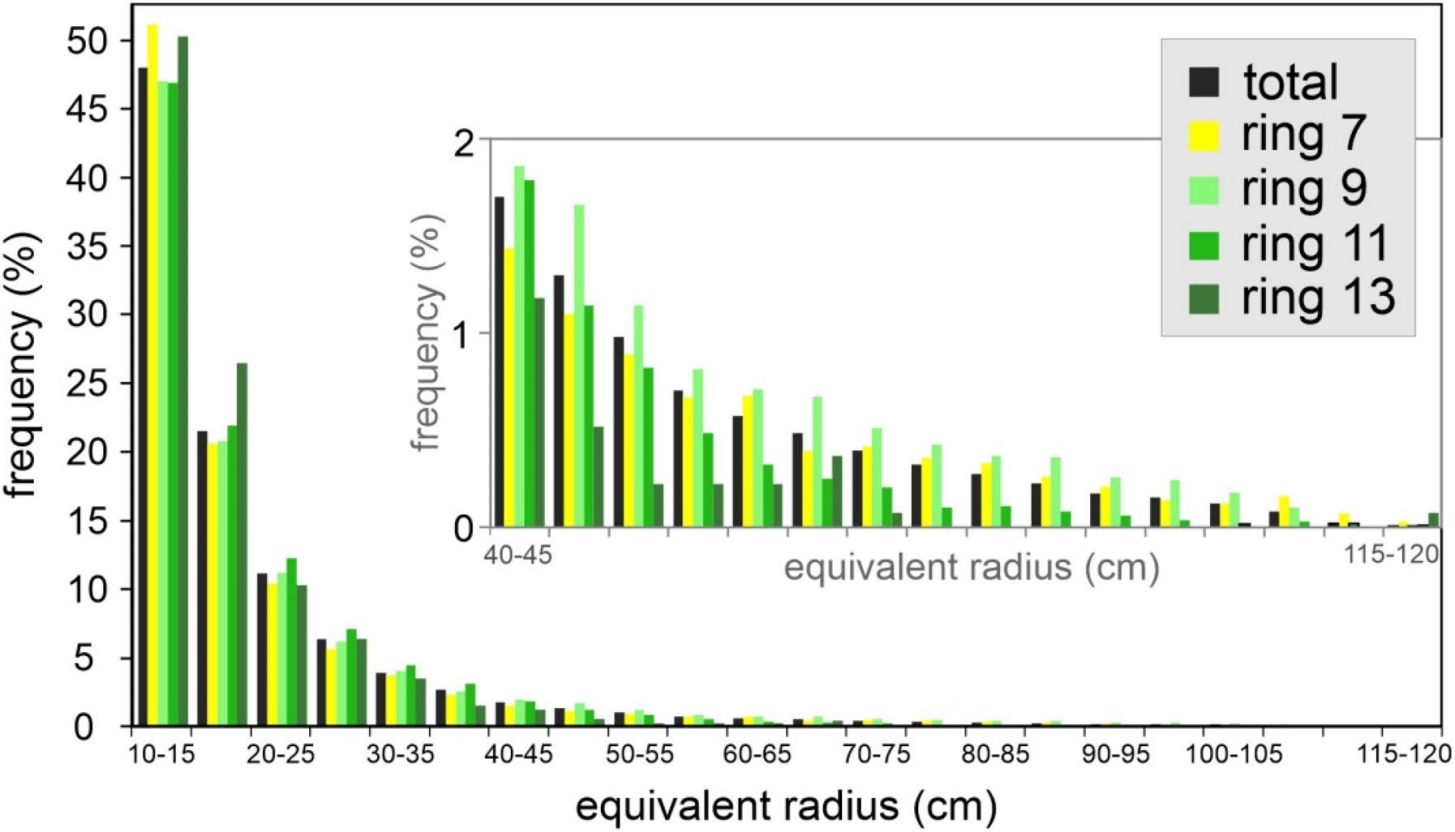
Spatter size frequency distribution of all mapped clasts (total) and the clasts mapped within selected rings (7, 9, 11 and 13). The size classes are defined by using 5 cm equivalent radius increment. The inset shows the enlargment of the diagram for size classes > 40 cm.

A further parameter investigated is the spatter clast size, here defined as equivalent radius of a circle reproducing the area of each spatter whose shape is assumed circular. Considering the entire deposit (152,228 clasts covering 29,000 m^2^), the equivalent radius ranges from 10 to 120 cm. We have analysed the size-frequency distribution of the total deposit and the clasts mapped inside the rings (Fig. 8). The size classes are defined by equal interval method by using 5 cm equivalent radius increment. The smallest size class is represented by the clasts with equivalent radius between 10 and 15 cm whereas the greatest size class is represented by the clasts with equivalent radius between 115 and 120 cm. Considering the total mapped spatters, the size frequency decreases with increasing size (Fig. 8) and, starting from the class size (15–20 cm), the frequency is reduced of 45–50% if compared with the previous class (Fig. 8). Such behaviour recurs in all rings and highlights a nearly constant ratio between the number of small and larger size clasts, regardless of the distance from the vent. Overall, the clasts with the smallest equivalent radius size (10–15 cm) represent 45–50% of all mapped spatters, while the size larger than 40 cm represents < 10%. The remaining 40–45% is composed by spatters with equivalent radius between 15 and 40 cm (Fig. 8).

## Discussion

### Erupted products volume estimations

The spatter clasts areal coverage of 29,000 m^2^, obtained through GIS-based automatic elaboration, allowed us to estimate the volume and the mass of the clasts scattered on the E flank of the volcano, emplaced during the early phase of the paroxysm. Assuming for the scattered spatters a thickness ranging from a minimum of 10 cm to a maximum of 20 cm (based on data presented in^18^), a density of 1300 kg/m^3^ (calculated from the spatters volume and mass data published in^18^) and associated error ± 20% on areal coverage, the volume estimation of the scattered spatter clasts results between 2.3 × 10^3^ and 7.0 × 10^3^ m^3^ and the mass between 3.0 × 10^6^ and 9.0 × 10^6^ kg.

Considering that abundant spatter clasts were emplaced also on the SW flank of the volcano and within the Sciara del Fuoco, the obtained values quantify likely about half of the volume/mass of all scattered spatter clasts emplaced on Stromboli flanks. From these considerations, it is reasonable to affirm that the total mass of the scattered spatter clasts deposited on the volcano flanks is between one and two orders of magnitude lower than the mass of the continuous spatter cover (1.4 × 10^8^ kg) calculated^18^ in proximal areas. Following these estimations, the total mass erupted as spatter clasts during the first phase of the paroxysm results more than double the mass of tephra erupted during the following phase associated with the rise of the eruptive column (5.9 × 10^7^ kg reported in^30^).

### Dispersion trends and morphological implications

**Figure 9:**
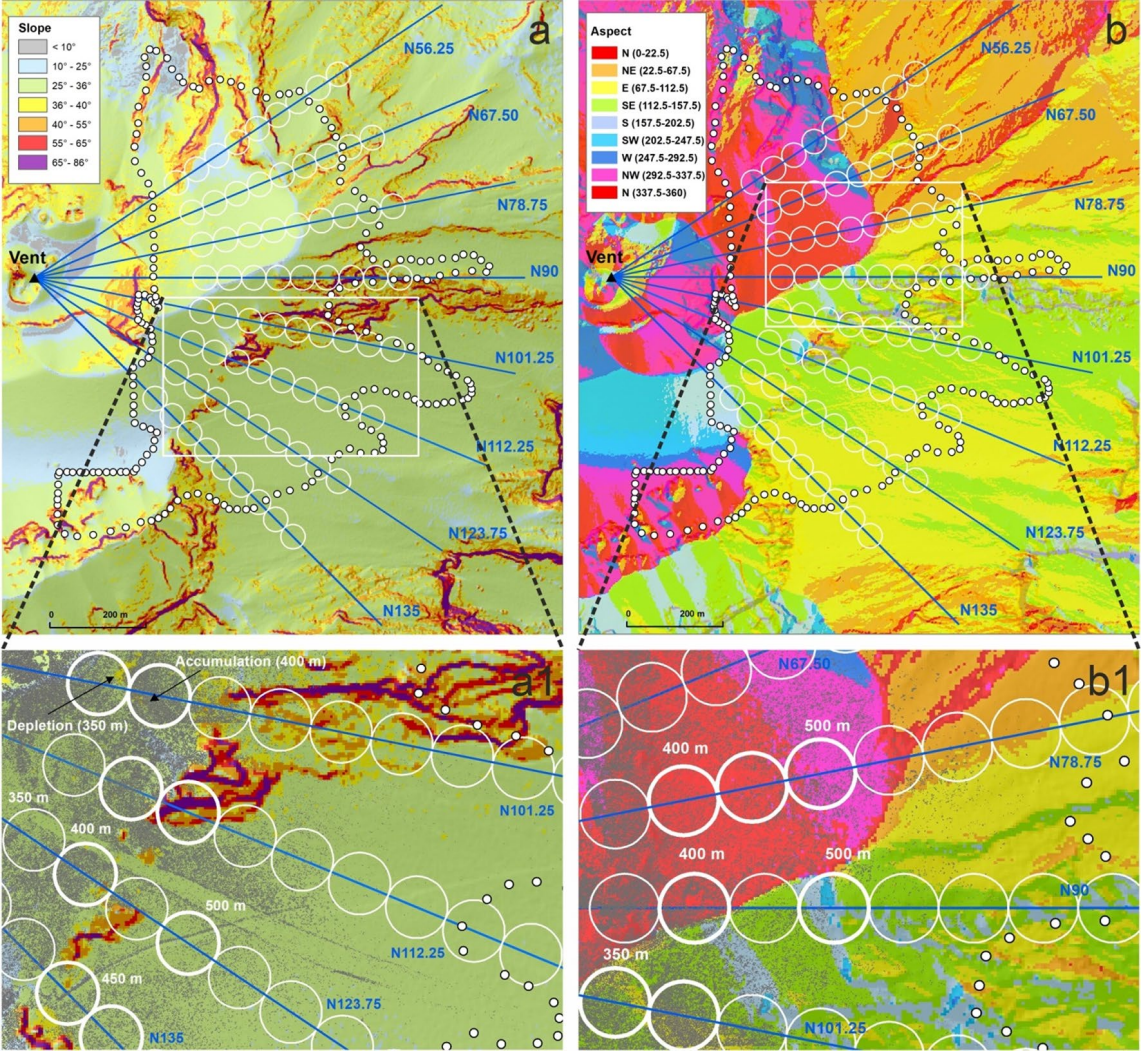
Slope (**a**) and Aspect (**b**) map with the zoom (**a1**, **b1**) of the respective inset (white rectangles). In overlay the investigated directions, the circles and the study area boundary. The maps are composed in ArcGIS 10.5 software (ESRI Platform).

The decreases of the SND and SCP along the directions (Fig. 7) are here analysed and discussed considering the two main morphometric parameters that control the morphology: Slope and Aspect (Fig. 9). Both are stored in matrix data and derived from the 2012 LiDAR Digital Surface Model of Stromboli following the same algorithm used in^34,35^. The matrix of the first morphometric parameter stores in each cell the value of the slope, expressed in angle, calculated considering the maximum rate of change in elevation over the distance between the cell and its eight neighbours. Instead, the Aspect matrix stores in each cell a value identifying the compass direction that the downhill slope faces for each location.

**Figure 10:**
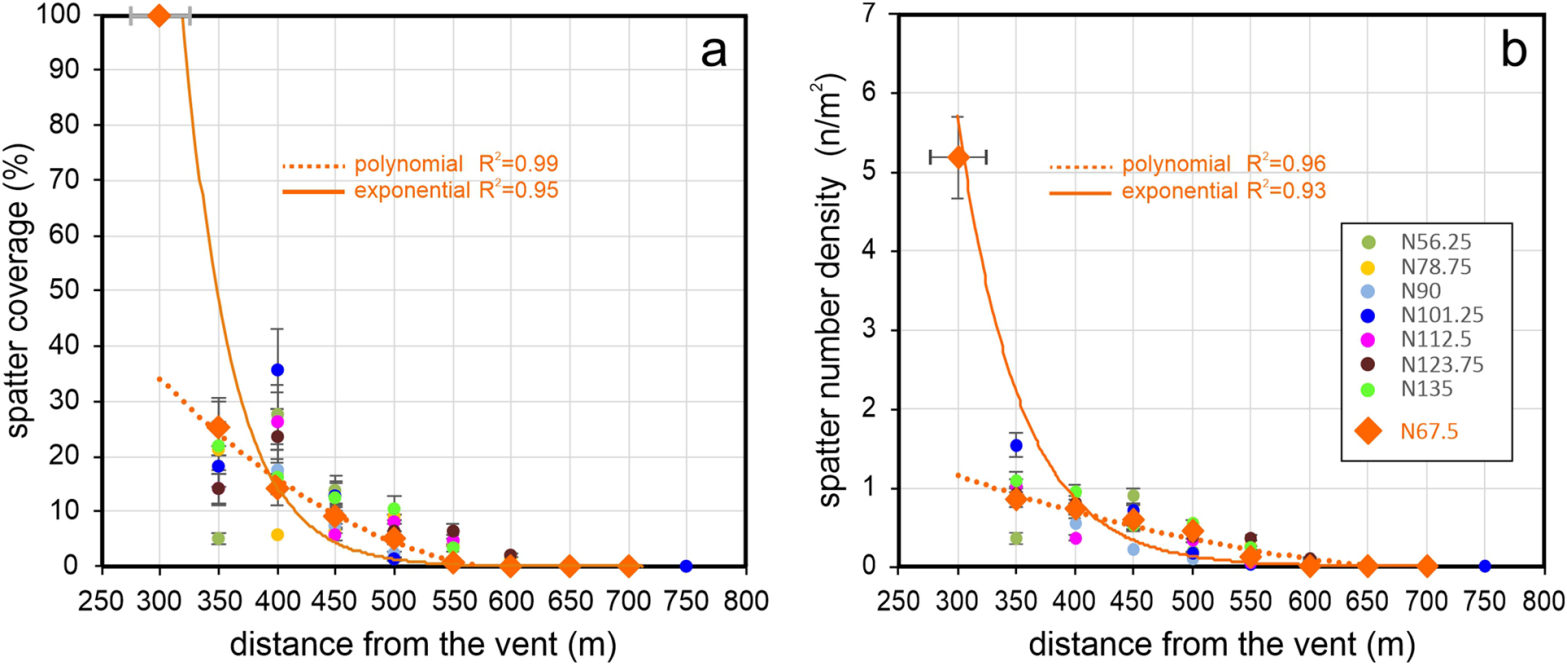
The best fitting trends for SND (**a**) SCP (**b**) parameters along the N 67.5 direction.

The progressive decreasing of both parameters observed only along the N67.5 direction (Fig. 7) involves morphologies characterized by slopes ranging from 25 to 36° (Fig. 9a) and mainly exposed to N-NW up to 550 m of distance from the vent. Only at distances ≥ 600 m the exposures change to NE-E (Fig. 9b). In order to better define such progressive decreasing of both parameters, best fittings trends were done. Considering the investigated circles (from 350 to 700 m from the vent), both parameters follow a polynomial trend (Fig. 10a,b). Instead, assigning to SND and SCP the limit values (maximum and minimum) and the corresponding distances from the vent, both parameters follow an exponential trend (Fig. 10a,b). In detail, the maximum value of SCP (100%) has been imposed, in according to^18^, at 300 m of distance from the vent (± 25 m). The maximum value of SND (5.18 n/m^2^), imposed at the same distance, has been derived correlating SND and SCP along the N67.5 direction. The minimum value of SCP (0%) and SND (0.002) has been imposed at 700 m, that is the distance derived from our mapping (Fig. 4).

Both fittings show that the decreasing of spatters dispersion along the N67.5 direction can follow different trends (polynomial or exponential), depending on the imposed constrains. Considering that along the N67.5 the slope and aspect parameters result nearly constant (slopes remain between 25 and 36° with exposures mainly to N-NW), the polynomial and exponential trends result probably more related to the eruptive dynamic and in-flight dynamic of spatter clasts rather than to the morphology. In the light of these considerations, a detailed analysis of the spikes observed in the other directions, if compared with the SND and SCP trends characterizing the N67.5 (Figs. 7, 10) is now presented.

The N56.25 direction shows spikes for both parameters in correspondence of a morphology characterized by significant changes in Slope (from 25 to 65°, Fig. 9a) and in Aspect (N, NW-W-NE, Fig. 9b). Along the direction N78.75, SND presents two small positive spikes (at 350 and 550 m from the vent), whereas SCP shows a marked negative spike at 400 m (Figs. 7b, 10b) and small spikes at 350 and 450 (both negative) and 500 m (positive). The negative SCP spike at 400 m could be explained considering that the previous circle (positioned at 350 m) contained a greater accumulation of spatter clasts that are slipped from very steep slopes (> 40°) positioned at 300 m from the vent. The small negative SCP spikes correspond to areas exposed to N, whereas a small positive spike is observed at 500 m where the Aspect parameter varies from N to NW (Fig. 9b1). In correspondence of these three small SCP spikes the slopes are the same (from 25 to 36°). Along the direction N90, the SND parameter shows two spikes: one positive at 400 m and one negative at 500 m (Figs. 7a, 10a). The positive spike affects areas where the slopes remain between 25 and 36°, while the Aspect varies from N to S-SE (Fig. 9b1). The negative spike affects areas where the high slopes (~ 45–50°) facing to SE-SW interfere with the spatter clasts deposition. As regards SCP, two only small spikes are visible. The positive one is positioned at 400 m where the Aspect is mainly to N (Fig. 9b1) and the slopes range from 25 to 36°. The negative spike is positioned at 500 m where morphologies with Aspect ranging from SW to SE and slopes similar to the previous ones characterize the areas involved. Only a very small portion of the respective circle presents slopes reaching 55°. Several spikes characterize N101.25 direction, but the most evident is positioned at 350 m for SND and at 400 m for SCP (Figs. 7, 10). To better investigate such spikes a more detailed analysis on slopes and exposures from 350 to 500 m has been done. The circle at 350 m (Fig. 9a1) shows an area free of spatter clasts, characterized by slopes ranging from 36 to 40°. This observation suggests a local remobilisation on this sloping ground, resulting in clasts depletion upstream and consequent strong accumulation downstream at 400 m (Fig. 9a1). The accumulation coincides with an evident positive deviation in SCP and a negative deviation in SND (Figs. 7, 10). The strong accumulation creates an almost continuous coverage so that the adjacent clasts are not distinguishable (Fig. 9a1). The Aspect is the same in both circles (E-SE). The subsequent negative SND spikes (at 500 m) is observed in correspondence to extensive areas, still exposed to the E, and characterised by very steep slopes (mainly > 55°) that interfere with the spatter clasts deposition as a barrier. The N112.5 direction shows one positive and negative SND spike at 400 and 450 m, respectively (Figs. 7a, 10a). The negative SND spike is positioned at 450 m where large areas with very steep slopes (55°) facing to SE are present (Fig. 9b1). Then, one marked positive SCP spike results at 400 m of distance (Figs. 7b, 10b) where the slopes remain between 25 and 36° but the exposures change from SE to SW (Fig. 9b). The N123.75 direction shows an evident positive SCP spike at 400 m (Figs. 7b, 10b) where the GIF is intercepted. This flow has remobilized the spatter clasts and generated an accumulation on the flow edges and on the flat trail track (Fig. 9b1). Moreover, small positive SCP and SND spikes are visible at 550 m and, probably, are related to the effect of trail tracks crossing the previous circle positioned at 500 m. Finally, the N135 is the only direction along which SCP and SND decreasing trends (Figs. 7, 10) result more similar to those observed along N67.5. Only very small positive spikes in SCP (from 400 to 500 m of distances) and SND (at 400 m) are observed along the N135 and they could be the effect of the trail track and the slopes > 40° characterizing the areas at 450 m of distance (Fig. 9a1).

All these analyses suggest that a progressive decreasing of the spatters dispersion with the distance (starting at 350 m from the vent) follows an exponential or polynomial trend unless the spatters deposition does not involves morphologies with slopes > 36°, and/or relevant changes of exposure. A mapping based on drone imagery was also applied by^13^ for reconstructing the spatial distribution of the ballistic projectiles generated by several explosions that occurred at Yasur volcano, during different periods. Although the work on Yasur’s is based on visual mapping of ballistics as geo-coded points it also concludes that the ballistics spatial density decreases with increasing distance from the vent, following a polynomial or exponential trend.

### Insights into preferential directions of the dispersion

The spatter clasts digital mapping obtained in this study (Fig. 3a) shows that the E flank of the volcano was affected by a heavy fallout of spatter clasts during the 3rd July eruption. Analysing the total number of spatters in the different angular sectors, two preferential dispersion directions are highlighted, one in sector **8** (N78.75–N90) and the other in sector **11** (N112.50–N123.75). The variation of SND and SCP parameters (Figs. 4 and 5) only partially confirms the occurrence of these two preferential directions. The rings **7**–**10** (300–500 m from the vent) are characterised by rather irregular variations of SND and SCP with no clear peaks in sectors **8** and **11**. Conversely the variation of the same parameters along the more external rings **11**–**14** (500–700 m from the vent) reproduces two peaks: one in sector **7** and the other in sector **11** (ring **11)**, and one in sector **8** and the other in sector **11**, for the subsequent rings (**12**–**14**). Considering the morphometric analyses presented in the previous section, the highly rugged terrain morphology of the proximal areas (300–450 m from the vent), generates effects of accumulation and depletion of the spatter clasts that, largely, influence the variation of SND and SCP parameters, overriding the primary distribution of the spatter clasts (defining as “primary” the distribution obtained without post/sin depositional phenomena). This primary distribution is obtained in distal zones, especially in the Rina Grande area (sectors **10**, **11**, **12**) where the morphology becomes less steep. Overall, all analyses above presented and discussed, suggest an evident preferential direction between N112.50 and N123.75 and a less marked one between N67.50 and N90, both likely related to the explosion dynamics.

### Insights into the clasts size distribution

The clasts size distribution of the mapped clasts is characterized by a rapid decreasing of the size frequency with the increasing size. Moreover, a nearly constant ratio between the number of small and larger size clasts is observed considering both the spatters mapped in the entire study area and the spatters mapped in each ring, regardless distance from vent. This behavior likely depends on to the eruptive and in-flight dynamics that prevent an increase or decrease in volcanic ballistics size with the distance as observed in other types of eruption^5^. However, the lacking of correlation between SND and SCP observed inside several rings (e.g. rings **8**, **9**, **10**) highlights clast size inhomogeneity in the different elements (**r**, **s**) of the single rings. Such inhomogeneity can be due to the post-impact slipping above discussed, but we cannot rule out that it is in part related to eruptive dynamics involving clustering of large (or small) spatters in discrete areas.

### Methodology: source data and processing

A UAV (unmanned aerial vehicle) survey was carried out on July 9^th^ with a long-range drone, a “Wingcopter Heavy Lift 178”. For image acquisition the UAV was equipped with a Sony Alpha 7R II full-frame camera with a 35 mm equivalent focal length. The flights were conducted with automated missions with flight altitudes of 300–350 m above ground level, the survey covered the whole island providing images at very high spatial resolution (8 cm), by using 60% front and 60% side overlap. The images were processed^36^ using the photogrammetry software Agisoft Metashape and subsequently imported and elaborated in a GIS environment (ESRI platform). In detail, the RGB drone imagery was ortho-rectified by using the 2012 Lidar-DSM (spatial resolution of 50 cm and vertical accuracy < 15 cm). The ortho-rectification is based on the Ground Control Points (GCPs) method and, in addition, takes into account the GCPs elevation derived from the DSM. We have selected a series of anthropic features, considered stable/unchanged during the time, visible both on DSM and drone imagery. Such features were selected to have a spatial distribution as uniform as possible to cover the entire image. In detail, we have used the centroid points related to the following anthropic features: shelter roofs, the building roof, the power plants and specific elements along the coast. The procedure of ortho-rectification was performed following 3 main and standard steps^37^: (i) matching of the source and reference point (GCPs) with the inserting of the relative elevation; (ii) running ofthe transformation equation; (iii) RMSE calculation. The very high spatial resolution of the imagery, visualised with an appropriate scale, allowed us to determine the dispersion area boundary of the visible spatter clasts, well recognizable thanks to a darker colour compared to the lithics. To automate the mapping spatter clasts procedure, a workflow was implemented within the GIS platform. The study area was divided into 35 tiles (160 m × 100 m) and each one was individually processed as follow: (i) extraction and storing of three channels R, G, and B as 8-bit images; (ii) the image channel that best visualises the pixels containing spatter clasts is clipped with the tile’s extent and analysed to identify the brightness values corresponding to pixels containing the spatter clasts; (iii) the brightness interval (minimum and maximum threshold) identified for each tile is used to select the corresponding pixels by querying the clipped image channel through GIS matrix operators; (iv) the selected pixels are automatically transformed into polygonal vector elements according to the raster-vector topology criteria^38^. At the end of the workflow, the polygon elements reproducing the boundary of spatter clasts have been organised in a geo-spatial database reporting unique identification code, tile number, areal coverage (m^2^), and coordinate x and y of the centroid (m). Finally, the resulting polygonal map was checked by overlapping it to the imagery and assigning a code to each polygon distinguishing the clast from a possible shadow or error. Considering the imagery cell size (8 cm × 8 cm) and the potential shadow, generated by a clast of one pixel, as a portion of the clast itself, the minimum detectable clast size corresponds to an area of 0.03 m^2^ and the error on clast area is 20%.

## Conclusions

A high-resolution mapping of the scattered spatter clasts emplaced on the E flank of Stromboli during the early phase of the 3rd July 2019 paroxysm is presented in this work. The digital mapping, obtained elaborating drone imagery with GIS-based methodologies, has identified 152,228 spatter clasts with areal dimensions ranging from 0.03 to 4.23 m^2^ and has reproduced the bi-dimensional geometry of each spatter clast as a geo-spatial polygon element. The mapped clasts are dispersed in an area of 0.407 km^2^, between N33.75 and N157.5 directions, and cover an extent of 29,000 m^2^ with an associated error of ± 20%. This data allowed us to estimate, for the first time, the volume of the scattered spatter clasts emplaced on the eastern flank, resulting between 2.3 × 10^3^ and 7.0 × 10^3^ m^3^. The zones of preferential emplacement lie in the sectors between N67.5 and N135 directions, where the clasts reach the maximum distance of 950 m from the vent (exactly in N90 and N101.25 directions) and the maximum number (precisely in the sector between N112.25 and N123.75). Two spatter clasts dispersion parameters, Spatter Number Density (SND) and Spatter Coverage Percentage (SCP), have been initially investigated through a slice of fixed angular width (11.25°) and, subsequently, through 25 m radius circles positioned every 50 m along a series of directions, starting at distance of 350 m from the vent. The first investigation highlighted the areas mostly affected by the deposition, whereas the second one has revealed, through best fitting analyses, the main trends that could describe how SND and SCP parameters vary with the distance from the vent in absence of consistent/relevant morphological changes. In such case, the spatter clasts dispersion decreases with increasing distance from the vent following polynomial or exponential trends. When the spatter clasts emplacement involves slopes > 36° (threshold above which the spatter clasts can start to slip) and/or relevant changes of terrain exposure, the parameters deviate from the trends previously mentioned, showing positive or negative spikes. In addition, deviations from the trends are also observed when the investigated directions intercept sliding phenomena. Overall, the analyses indicate the presence of two preferential directions of the spatters distribution, one between N112.50 and N123.75 and the other one, less marked, in the N67.50-N90 directions. Investigations on clasts size distribution highlight a rapid decrease of clasts size frequency with the clasts size increasing and a constant large/small clasts ratio regardless the distance from the vent. All these results show that the novel methodological approach presented here allows to derive in an efficient way and at very high spatial resolution a larger amount of data and information on ballistic fallout deposits that it is impossible to obtain in a reasonable time with classic field surveys, especially in areas not accessible. At Stromboli the ballistic fallout affects the areas usually reached by volcanologists for field survey and zones visited by guides and tourists before the 2019 crisis and during the paroxysms can affect the inhabited areas on the coast. Considering that most of the data derived in this study are preparatory/essential to develop subsequent robust probabilistic hazard maps, this work provides a useful contribution for further studies aimed at reducing the risk of ballistic showers at Stromboli and also in other areas characterised by similar volcanic activity.

## Data Availability

The datasets used and/or analysed during the current study available from the corresponding author on reasonable request.
